# Legal Frameworks and Controls for the Protection of Research Animals: A Focus on the Animal Welfare Body with a French Case Study

**DOI:** 10.3390/ani11030695

**Published:** 2021-03-05

**Authors:** Elisa Codecasa, Patrick Pageat, Míriam Marcet-Rius, Alessandro Cozzi

**Affiliations:** 1 Animal Experimentation Service, IRSEA (Research Institute in Semiochemistry and Applied Ethology), Quartier Salignan, 84400 Apt, France; 2 Responsible for Innovation, IRSEA (Research Institute in Semiochemistry and Applied Ethology), Quartier Salignan, 84400 Apt, France; p.pageat@group-irsea.com; 3 Animal Behaviour and Welfare Department, IRSEA (Research Institute in Semiochemistry and Applied Ethology), Quartier Salignan, 84400 Apt, France; m.marcet@group-irsea.com; 4 Research and Education Board, IRSEA (Research Institute in Semiochemistry and Applied Ethology), Quartier Salignan, 84400 Apt, France; a.cozzi@group-irsea.com

**Keywords:** laboratory animals, legislation, animal welfare, animal welfare body, French body

## Abstract

**Simple Summary:**

Worldwide, the conditions governing the use of laboratory animals are based on culture and traditions. Applications of the principle of the three Rs—replacement, reduction, and refinement—aim to foster humane and responsible science and minimize animal harm. In this paper, the European system regulating laboratory animal welfare is examined since it is one of the strictest such systems in the world. Growing public concern over the use of animals for research animal use and the importance of high research animal welfare to the outcomes of studies have pushed institutions to strengthen their protocols for research activities involving animals. The animal welfare body (AWB) is a type of regulatory structure responsible for protecting animal well-being in each stage of experimental research programs. It is a local structure that fulfills oversight and advisory functions on animal housing conditions and care and research procedures involving animals while providing support for the implementation of legal requirements. Since this structure is an innovation and examples of its functioning are scarce, a French case study is described here. Information on AWB members’ functions, tasks and goals is discussed by examining the body’s activity in a French research institute. The purpose of this work is to add knowledge in this field and share information on the scientific community’s commitment to welfare progress for laboratory animals.

**Abstract:**

In recent years, efforts have been devoted to improving the welfare of laboratory animals. Scientific progress and growing concerns over animal harm have pushed institutions to strengthen their laws to make science more humane and responsible. European Directive 2010/63/EU makes it mandatory for breeders, suppliers and users of laboratory animals to have an animal welfare body (AWB) to prioritize animal welfare and harmonize experimental standards while reassuring the public that research is being carried out appropriately. Based on application of the three Rs (refinement, reduction and replacement), these bodies provide staff with oversight and advisory functions to support compliance with the legal requirements on both animal housing and project realization. This review aims to present the legal measures protecting research animals, with a focus on European AWBs. The review explains how the mission of AWBs includes development of environmental enrichment programs and how animal training generates benefits not only for animal welfare but also for the research work environment and research quality. A French case study is conducted to provide the scientific community with an example of an AWB’s functioning and activities, share its achievements and propose some perspectives for the future.

## 1. Introduction

The legal framework for the protection of research animals has evolved over time in Europe and in the rest of the world. Advancements have been made in the field of science, with related developments of expertise and good practice in research animal protection, and increases in societal concerns over laboratory animal use have contributed to the enactment of and updates to laws [1,2,3]. In recent decades, animal welfare has become a policy priority and provisions on the issue have appeared in legislation of all types, including treaties [4,5], laws [6], conventions [7] and directives [8]. Since the globalization of science has increased the heterogeneity of legislative frameworks, there is an aim to harmonize standards and create a level playing field for scientists [9,10] while providing reassurance to the public that research is being carried out with proper checks and balances [1]. To achieve this, current regulations provide minimum standards for laboratory animal housing and care, fostering respect for and improvement of the three Rs (reduction, refinement and replacement) [3]. Guidelines [11], codes of practice [12] and non-statutory recommendations are available to enable institutions to better adhere to and implement the legal requirements. In addition, structures operating at different administrative levels are essential. Competent authorities and national and local committees are all involved in the protection of animals through different missions, such as the authorization of establishments and project evaluation and authorization [9,13]. The animal welfare body (AWB) is one of these regulatory bodies, with which all laboratory animal breeders, suppliers and users must engage in their own establishment with the primary task of focusing on animal welfare issues [8]. The AWB is the name of this group in Europe, but similar structures exist abroad: for example, the US has the Institutional Animal Care and Use Committee (IACUC) [11]. Depending on the country, the AWB’s organization and functioning can vary, but its purpose is the same: to give animal welfare issues the highest priority. Its main tasks are to support staff regarding animal acquisition, accommodation, care, use and rehoming schemes; to monitor the development and outcome of projects; to foster a climate of care; and to provide tools for the application of developments in replacement, reduction and refinement [8]. In this article, the authors aim to describe the AWB in depth as the crucial organ engaged in the protection of laboratory animals. To contextualize the legal environment, information on worldwide laws regulating research animal use is first provided with a focus on the European system; in this section, a description of the ethical principles on which regulations are based is provided. The manuscript continues by presenting the main actors and tools set up in the EU to guarantee the implementation of the laws; the AWB is therefore described in detail, and a section is dedicated to the French version of the body. As far as we know, there are few practical examples of animal welfare bodies discussed in the literature; thus, we report on the case of a French research institute.

## 2. State of the Art of Regulatory Frameworks for Research Animals

### 2.1. A Global Overview of Laws and Standards Regulating Animal Experimentation

The history of the use of animals to advance human knowledge is long. To control this activity and minimize harm to animals, regulatory measures have been developed worldwide [1]. This is consistent with the principle that members of the scientific community who use and produce animals in laboratories must at minimum assume responsibility for the animals’ welfare [10]. The very first legislation in the world that was specifically directed towards the protection of animals used in science dates back to 1876 in the United Kingdom with the Cruelty to Animals Act of 1876 [14]. Other countries, such as Australia in 1883 [15], Denmark in 1891, Germany in 1933 and Sweden in 1944 [1], followed suit in signing laws to promote animal welfare. Societal laws, culture and traditions and the history of laboratory animal science in each country have influenced the conditions governing this sector [10]. This explains why the regulatory regimes are longstanding and sophisticated in some countries but poor, absent or being improved in others.

#### 2.1.1. US, Canada and Latin America

In the US, the primary regulation intended to improve the treatment and well-being of research animals is the federal Animal Welfare Act (AWA) of 1966, overseen by the United States Department of Agriculture (USDA). The AWA provides standards for the use of laboratory animals, but since 2002, it has specifically excluded birds, rats and mice bred for research [6]. A separate piece of legislation, called the Health Research Extension Act, was passed in 1985 and covers all vertebrates used in research, testing and education—including the mice, rats and birds excluded under the AWA—if the work is funded by the Public Health Service (PHS) [16]. The Guide for the Care and Use of Laboratory Animals sets the standards for care and housing that must be provided for animals in PHS-funded studies [11]. The Canadian system, unlike the US system, does not have legislation directly governing the care and use of animals in research, which are instead based on customs and practice. The central feature of the Canadian system is the Canadian Council on Animal Care, which was formed to support universities and government departments involved in animal-based science [17]. With the exception of a few nations, Latin America is a region that lacks legal frameworks or governmental infrastructure for the oversight of research animal use. The most developed policies exist in Brazil, Mexico and Uruguay. In Brazil the current law 11.794 was enacted by federal decree 6899 in 2009. Its implementation is ensured by the National Council on the Control of Animal Experiments and based on guidelines and technical guidance documents on the use of animals in teaching, research or testing. In Mexico, the law NOM-062, Technical Specifications for the Production, Care and Use of Laboratory Animals, was enacted in 1999. It is based on internationally accepted documents as the Guide for the Care and Use of Laboratory Animals [11]. In Uruguay, the law 18.611 regulates the animal use in experimental activities, teaching and scientific research in collaboration with the National Commission for Animal Experimentation. In other countries, efforts have been made to adopt international standards and enact laws on the use of animals in testing and teaching [18].

#### 2.1.2. Pacific Rim

In Australia, regulatory frameworks on laboratory animals are decentralized, with each state largely under the control of its own policy [19]. Despite this, all laws are based on the Australian Code of Practices for the Care and Use of Animals for Scientific Purposes, which was enacted in 1969 and last amended in 2004 [20]. In China, a hierarchical system gives the Ministry of Science and Technology responsibility for the Administration of Laboratory Animals, while local governments create central, regional and institutional levels of control. Japan has a decentralized model based on the Law for the Humane Treatment and Management of Animals of 1973 [10], while voluntary guidelines such as the Act on Humane Treatment and Management of Animals are provided by various organizations [21]. Institutions exercise self-regulation following guidelines provided by the Ministry of Education. Singapore, Thailand, Indonesia and Malaysia have different legislative statuses concerning the research animal sector, with the countries’ regimes based mostly on implementation of the “Guidelines on the Care and Use of Animals for Scientific Purposes”, published in Singapore in 2004. The Indian Parliament passed the Prevention of Cruelty to Animals Act in 1960, followed by the creation of the Committee for the Purpose of Control and Supervision of Experiments on Animals, which is in charge of developing its “Guidelines for Laboratory Animal Facility” [10].

#### 2.1.3. Africa and Middle East

Animal research is performed in African and Middle Eastern countries, but many of them have neither legislation nor guidelines; despite this gap, institutions are adapting standards and practices to improve laboratory animal protection [10].

#### 2.1.4. The European Legal Framework

In Europe, Directive 86/609/EEC of 1986 [22] was one of the first examples of common legislation to set standards for housing and caring for laboratory animals across the member states of the former European Economic Community (now the European Union) [3,23]. In 1986, the Council of Europe published the European Convention for the Protection of Vertebrate Animals used for Experimental and Other Scientific Purposes [7]. Starting in 2002, a process of revising European animal experimentation was undertaken [3,23], resulting in Directive 2010/63/EU, which has regulated this activity in Europe since 2013 [8].

The reasons that prompted the EU Parliament to revise the former regulation were related to the need to give animal welfare a higher status, foster ethical principles of care and use and provide a more standardized legal environment across member states [3]. Two historic agreements also contributed to the improvement of the previous law: the Treaty of Amsterdam of 1997, which established the status of animal welfare [4], and the Treaty of Lisbon of 2007, which strengthened the status of animal welfare with an article requiring that “in formulating and implementing the Union’s agriculture, fisheries, transport, internal market, research and technological development and space policies, the Union and the Member States shall, since animals are sentient beings, pay full regard to the welfare requirements of animals, while respecting the legislative or administrative provisions and customs of the Member States relating in particular to religious rites, cultural traditions and regional heritage” [5] (p.41). In addition, changes in science and growing public concern over the use of animals in research [23,24] motivated the EU Parliament to reinforce and adapt its laws. The Directive 2010/63/EU contains 59 introductory recitals, 66 effective articles and 8 annexes [3,13]. The articles are organized into six chapters that regulate, among others, the field of application and the three Rs principle, minimum standards for experimental facilities, requirements for responsible parties and the role of different entities in the implementation of the law. The robustness of the directive derives from its large scope in terms of coverage of types of animals (vertebrates, cephalopods and later fetal and larval stages of vertebrate animals) and science (research, testing and teaching). Furthermore, all phases of animal life are regulated due to personnel and institution licenses and the provisions for animal welfare responsibilities [3]. Despite this, since its implementation, Directive 2010/63/EU has been publicly criticized by groups arguing for increased protection of animals such as the 2015 Stop Vivisection Citizen’s Initiative, which aimed to nullify the directive and pushed for adoption of legislation that would fully phase out animal experiments [25]. The political consequences of the initiative were negligible, but the EU committed itself to promoting and harmonizing the three Rs principle and to enhancing the protection of research animal welfare [3,26]. With the new directive, Europe took a leading role in research and development of new non-animal tests by introducing a series of measures that strengthen the evaluation of the need for animal use in each case [26]. In the transposition of the directive, control systems and specialized organisms have been set up by member states with oversight functions for each aspect of the chain of animal involvement, such as inspection of facilities and evaluation of experimental projects before they start [3,9].

### 2.2. The Three Rs Principle in Legislation

The use of animals in research has been the subject of public, scientific, ethical and philosophical discussion for hundreds of years [24,27]. In 1959, Russell and Burch proposed the concept of the three Rs—replacement, reduction and refinement—in their book The Principles of Humane Experimental Technique [28]. The term “replacement” was used to define “any scientific method employing nonsentient material which may, in the history of experimentation, replace methods which use conscious living vertebrates”; the term “reduction” represented a “reduction in the number of animals used to obtain information of a given amount and precision”; and “refinement” meant “simply to reduce to an absolute minimum the amount of stress imposed on those animals that are still used” [28]. Currently, the three Rs principle has become synonymous with measures to improve laboratory animal welfare, and the principle is now internationally accepted as the ethical framework under which experiments should be conducted [29]. The three Rs can be directly included in legislation [8] and guides [11], or their adoption and implementation can be implicit. For example, in countries where laboratory animal welfare laws have yet to be established, the three Rs are understood and applied [10]. In Europe, the former European Directive 86/609/EEC did not explicitly mention the three Rs, but at the same time, it required member states to put into practice much of their content [29]. The updated Directive 2010/63/EU had the specific aim of improving the three Rs principle, requiring its systematic application in all interactions with laboratory animals. Alternative methods and the refinement of animal housing, care and experimental procedures were highlighted to eliminate or reduce any possible pain, suffering, distress or lasting harm to animals to safeguard their well-being [22]. The three Rs have been actively promoted by the laboratory animal science community, which assumes that their adoption is crucial for the quality of outcomes [1,30]. In vitro or in silico methods such as the use of tissues and cells and computer models have been developed to replace animals. In addition, computer programs help to better define the size of the animal sample to minimize the number of subjects per experiment in line with scientific aims [29,31]. The animal experimental model is considered to require constant improvement to be more reliable and informative [32]. In this context, the refinement not only of protocols but also of husbandry and handling of breeding stocks has received more emphasis; indeed, if good measures have a positive impact on laboratory animals’ lives, the reduction of their stress responses may, at the same time, ameliorate bias in experimental results [29,30,33]. The three Rs framework has also contributed to providing transparency and some reassurance for members of the public who are concerned about laboratory animals through the standardization of measures for the protection of animals and their welfare [1].

## 3. Legislative Actors and Tools for the Protection of Laboratory Animals

Regulatory frameworks governing laboratory animal science operate through different structures, such as competent authorities (governments), ethics committees (ECs) and other bodies aimed at verifying good compliance with legal requirements and protecting animal welfare [9]. Practical tools such as application forms, checklists or guidelines are provided and used to ensure, improve and standardize global monitoring. In this section, a brief description of the European system is first reported, followed by an entire section on the main regulatory structure considered in this article: the AWB.

### 3.1. European Policy Functioning

The competent authority is the structure designated by a member state to carry out the obligations arising from Directive 2010/63/EU [13]. In Europe, this role is played mostly by the Ministry for Agriculture and Environment (the Home Office, in the case of the UK government) and sometimes the Ministry for Health, Education, Science and Innovation [9,34]. In some countries, this responsibility is shared between two or more ministries [9]. Moreover, according to the directive, each member state should establish a national committee. This structure collaborates with ministries and with other agencies (e.g., the National Committee for Ethical Reflection on Animal Experimentation in France [35]) to promote coherence and consistency on matters relating to the protection of research animals [36]. In addition to these central organisms, European policy acts at a more local level through specific structures designated by the competent authority to perform activities focused on the evaluation and authorization of projects and the inspection and authorization of establishments, including assessments of competences, the welfare and care of animals and the overall implementation of the three Rs [9]. Concerning the realization of projects, member states shall ensure that experiments are not carried out without prior authorization from the competent authority [8]. Some member states, such as Germany and the UK, assign responsibility for project evaluation to the same competent authority; others delegate this responsibility to committees that can be national, regional or local. Belgium and France, for example, have ECs attached to a single establishment [9,34]. To obtain project licenses, researchers are asked to fill in an application with the project proposals and all elements of the scientific work, including details about the three Rs [37]. Member states can use common templates such as the one developed in France by the Ministry of Research [13]. In member states where the competent authority oversees the project authorization process, prior evaluation by the research establishment is often requested. In countries involving other organizations, such as France, the applications are first evaluated by an EC and subsequently sent to the competent authority for a second evaluation and the consequent authorization [34,38]. Inspections of facilities of breeders, suppliers and users are normally performed by veterinary authorities in charge of verifying the legal standards of housing and practice [39]. This procedure allows establishments to be authorized by and registered with the competent authority [8]. Several tools, such as standard checklists, are available to assist institutions in conducting the inspections and to promote their consistency [40]. Staff education and training requirements, including attainment and maintenance of competence, are also evaluated during inspections [9]. Some training bodies have developed tools for competence assessment, such as the EU guidance document on the Education and Training Framework [41]. In addition to these provisions, oversight functions and guidance on matters related to animal welfare and daily application of three Rs principle are required by law. The directive delegates this responsibility to a set of institutional entities that, due to the nature of their tasks, are collectively called the animal welfare body [8].

### 3.2. The Animal Welfare Body

One of the novelties introduced by Directive 2010/63/EU was the creation of new organs such as institutional AWBs [3]. According to the legislation (recital 31 and articles 26 and 27), an AWB must be set up in every institute that keeps, breeds and uses animals for research. AWBs aim to protect laboratory animals, providing them with the best possible living conditions. In doing so, their main tasks are, as a minimum, to advise staff on animal welfare issues regarding acquisition, accommodation, care and use; to follow the scientific projects and their outcomes, taking into account the effect on the animals used; to provide tools for the implementation of the three Rs; to establish and review internal operational processes and to advise on rehoming schemes for retired animals [8]. According to the law, an AWB must include at least the person or persons responsible for the welfare and care of animals and, if the animal user is a research institute, a scientist; additionally, the members receive input from a designated veterinarian. Members should include people with communication and educational skills and have an adequate level of knowledge and expertise in a number of key areas: legislation, animal ethology, husbandry, care, health, enrichment practices, welfare assessment and each of the three Rs [36]. Regarding practical functioning, AWBs should establish their own code defining the responsibilities of each member, the procedures followed and the frequency of meetings [42]. All interventions and decisions must be recorded and available for scrutiny during inspections [8]. AWBs are supervised by the competent authority while it can obtain advice and support from the national committees [36]. Structures like the EU’s AWB exist worldwide with different names, such as the Animal Care Committee (ACC) in Canada [17] or the IACUC in the US [43]. However, these local committees can be organized differently and often address both issues related to the ethical reviews of proposed research and animal welfare standards. To summarize, the AWB plays a fundamental role for both animals and science. It provides assurance to the research establishment by taking a lead role in promoting animal welfare and implementation of the three Rs, improves communication between scientists and animal care staff and influences management to ensure the availability of suitable resources for good science and welfare [36].

#### Relationship between AWBs and Ethics Committees

According to Directive 2010/63/EU, responsibility for project evaluation is not assigned to AWBs but to ECs. These structures should therefore be considered separate, and both should be set up by the same research establishment. In the French system, differences related to status, functioning and composition are reported. Although both entities are related to the same national committee, AWBs belong to the Ministry of Agriculture and Food, while ECs belong to the Ministry of Research [44]. Consequently, their registration processes differ: while the composition of an AWB is declared on the establishment’s own license application, the EC is registered through a different form through which it states its commitment to applying the principles of the national charter of the National Committee for Ethical Reflection on Animal Experimentation [45]. Moreover, while AWBs must be internal to the institute, ECs can be internal or external as long as members meet punctually for project evaluations; ECs are thus autonomous and independent from institutes, while AWBs instead have a strong relationship with the institute administrations. Finally, concerning the composition, AWBs should include at least two members, while ECs must contain at least five members to allow the expression of diverse views [8,45]. However, beyond these differences of configuration, the missions of AWBs and ECs can often be similar or overlap depending on each country’s policy. Thus, while in France, only ECs are designated for the evaluation and authorization of experiments, in Spain, AWBs can be named Ethics Committees for Animal Experimentation and defined as Authorized Bodies for project evaluation [13]. In other countries, such as Italy and the Netherlands, the two bodies are separate, but the tasks for obtaining project licenses are shared in a well-established way. Regardless of the strategy adopted, the missions of AWBs and ECs are complementary, and AWBs may be involved in some parts of the project evaluation process to achieve the best possible results in both laboratory animal protection and research work [42].

## 4. The French AWB in a Research Institute

European Directive 2010/63/EU was transposed into French law on 1 February 2013, with the Decree 2013-118 and its related five Arrêté (articles 214-87 to 214-138 of the Rural Code) [46]. The Arrêté laying out “the conditions for authorization, design and functioning of breeders, suppliers or users of laboratory animals” contains a paragraph on the AWB, whose French name is “Structure chargée du Bien-Être Animal” (SBEA). From an administrative point of view, the SBEA is attached to the Management of Population Protection departmental subdivision, which is in charge of its authorization and monitoring. The composition of the SBEA, including the name of the animal welfare officer, is provided during the application for the establishment’s authorization. Since in France, this authorization has a duration of six years, the SBEA’s membership may not change for this whole period, although its modification is possible by informing the authorities about new members. To better clarify how this structure operates, we propose a practical example provided by the SBEA of the Research Institute for Semiochemistry and Applied Ethology (IRSEA) currently operating in Southern France. The particularity of this institute is that it is specialized in the area of interest here, which is research on animal behavior and welfare, among others. Thus, refinement measures aimed at limiting animal stress, fear or aggressiveness to allow high-quality studies represent a primary research objective of the institute and the overall approach of the organization to animal welfare; in our case, these specific measures are therefore implemented both to comply with legal requirements and to meet research needs. This dual purpose reinforces the institute’s commitment to animal welfare issues, giving the SBEA a key role in the achievement of its goals. This section is divided into several parts: after a brief presentation of the research institute, the composition and functioning of the SBEA are described; examples of its mission are then presented, and the main challenges it faces and its perspectives towards the future.

### 4.1. The Research Institute in Semiochemistry and Applied Ethology

The IRSEA is a private research institute dedicated mainly to the study of the behavior of animals and humans, their interactions and especially their chemical communication. The aim of the institute is to study and understand the mechanisms underlying these functions and to develop methods to improve behavioral and interaction management and welfare. To conduct its research, the institute houses several species, such as cats, dogs, pigs, rodents and rabbits, in an experimental facility.

Departments specializing in different fields, such as fundamental and applied ethology, animal welfare, biology, chemistry and chemical ecology, collaborate to achieve the company’s goals and provide other services related to animal population management. Among them, the animal experimentation platform is a service provided by a team of animal keepers, technicians and engineers in charge of the establishment’s maintenance and the daily care provided to all the hosted species. In addition, the service plays a key role in coordinating research in collaboration with departments when they need animals, equipment and personnel for their studies.

### 4.2. The IRSEA’s SBEA: Membership and Functioning

At the IRSEA, the SBEA includes the director of the institute, two researchers (both heads of research departments) and the veterinarian in charge of the health and welfare of the animals housed. All the members are scientists; thus, if one of them is directly involved in an experimental procedure to be assessed, the others can take over that person’s role to limit any conflicts of interest [42]. Due to the institute’s research field, the team has knowledge and experience in animal ethology, welfare, ethics and legislation. According to a report from the commission to the EU Parliament and the Council (2020), the members’ functions are also divided into subgroups to ensure that all tasks are covered efficiently and effectively. Thus, in our example, the designated veterinarian is also the head of the animal experimentation platform. This allows the veterinarian to directly and regularly monitor the animals’ conditions, focus the attention of care staff on animal welfare matters, delegate tasks and keep the other SBEA members informed. An internal code defines the overall operating mode relating to the entity’s missions and the relationship with staff, the EC and external oversight authorities. This code is public and validated by the administration. All the SBEA’s activities and decisions are recorded, signed and kept for at least 5 years, as they may be requested during inspections. To complete the bureaucratic work, an annual report is finally written to summarize the aims achieved and challenges encountered and to define the goals for the following year.

### 4.3. The IRSEA’s SBEA: Tasks and Goals

Since the SBEA’s work deals with animal welfare issues in both routine maintenance and project periods, its description is provided with the information grouped into these two categories.

#### 4.3.1. The Role of the SBEA in Routine Maintenance of Laboratory Animals

At the IRSEA, the SBEA and the animal experimentation platform collaborate to advise and provide staff with technical-scientific support in their day-to-day mission. Based on the three Rs principle, refinement of the animal housing systems, care and handling is the common thread of the body’s strategy. Maintaining a good attitude towards animals among personnel is the first principle to be applied as part of this strategy. Considering that animals may react with fear when staff enter rooms or clean cages [47], calm gestures and a low voice are recommended; therefore, knocking on facility doors is encouraged to avoid surprising animals due to sudden staff entries. Considerable attention is given to environmental enrichment programs such as species-specific opportunities within the environment to enable animals to express a diversity of species-appropriate and desirable behaviors [48]. According to Hosey G et al. (2009), environmental enrichment can be divided into five categories: food-based, physical, sensory, cognitive and social enrichment. The SBEA’s mission is to develop these types of enrichment together with socialization and training activities to improve the lives of animals, avoiding the onset of problems such as boredom, stress or stereotyping. Concerning physical enrichment, the enclosures contain means to allow animals to explore and play but also hide or escape from mates. An example is represented by the catteries, which are designed as three-dimensional spaces boasting shelters and bridges, corners to hide, separate areas for food, rest and elimination and enough resources for all individuals [49]. For pigs, the pens contain straw bedding to allow animals to root and rest comfortably. For dogs, enclosed boxes are used at night, while an outdoor green park is available and equipped with shelters to host animals during the day. Finally, the rabbit and mice cages are enriched with houses, tunnels, nest materials and chip bedding, while an additional floor enclosure has been adapted to provide rabbits with a larger and more complex space. For all species, additional items such as commercial and handmade toys are supplied and renewed regularly. Food-based enrichment is managed by hiding food in holed containers, while for sensory enrichment, synthetic analogs of pheromones are diffused in accordance with ongoing research projects to avoid affecting behavioral studies [50]. Concerning social and cognitive enrichment, animal training activities are also included in their management. To meet the social needs of the species housed, the IRSEA’s animals are kept in groups; in addition, programs aimed at socializing animals to humans are a main part of the care staff’s schedule. To this end, play and care activities and training based on positive reinforcement are planned. In play sessions, the use of feathered rods and cardboard for cats, balls for dogs or plastic rings for pigs is alternated with active games. For the latter, agility runs with obstacles and tunnels made with recovery materials can be used outdoors with dogs, while an indoor corridor is set up and adapted to enable pigs to exercise and maintain a healthy weight. In addition, cognitive activities, during which animals solve problems to reach a reward, are also used. During care activities, animals are brushed and gently cleaned to create and maintain a positive human–animal relationship. Finally, activities are organized to habituate animals to handling, to performing behaviors or to facing a new environment while offering them stimulation. For cats, training in the use of carriers or restraints for blood collection represent some of the most typical sessions, while for pigs, the activities focus on habituating the animals to climbing spontaneously on the scale or to remaining calm in the restraint sling. Food rewards for cats or for pigs are combined with vocal appreciation to both reinforce positive behaviors and associate novelty with an enjoyable time. As the safeguarding of laboratory animal welfare depends in a large part on the professional skills of the staff in charge of their care, the monitoring of competences is among the SBEA’s key priorities. To this end, training courses are regularly organized; these meetings allow training of staff, discussion of problems and sharing of ideas. Additionally, to ensure that activities are conducted regularly and with homogeneous approaches, a document is prepared and updated by the SBEA to describe the procedures and their frequency during the week. Videos are also used so that people can review the techniques multiple times. Beyond these functions, audits of facilities are organized once a month to verify compliance with internal operating procedures. Normally, these controls are only performed by one member, who is different each time to ensure different views on welfare standards. During the audit, a prefilled form is used to record all information. Like this, the methods adapted for each laboratory species are continuously being developed by the IRSEA team to more effectively evaluate the animals’ welfare level, share the results with personnel and plan improvements [51]. Due to these audits, decisions have been made to upgrade animal housing. For example, as the SBEA considered the living space for pigs too small, additional large pens were adapted to allow animals to exercise more and choose to flee their mates to avoid conflicts. The SBEA can also intervene upon request for specific welfare problems on which the intervening member has a high level of related experience. In this case, the SBEA’s approach is similar to a medical one: it visits the group of animals, diagnoses the problem and its triggers and suggests a treatment accompanied by a follow-up. Aggressive behaviors between pen mates are a common situation. If aggressiveness is related to scarce resources or mistakes made in their provision, corrections may solve the problem. However, in cases where fighting becomes very serious, the SBEA may decide to rearrange groups; integration programs are therefore defined, explained to the staff and monitored. As already explained, all SBEA activities are recorded; while this phase is crucial for the effective monitoring of decisions, at the same time, the recordings can support future choices. A timely and precise flow of information is guaranteed by the SBEA for all personnel so that everyone can feel involved in animal welfare issues. Finally, a public calendar of the SBEA’s interventions is available to allow care staff to prepare questions in advance if necessary.

#### 4.3.2. The Role of the SBEA in Experimental Procedures

At the IRSEA, studies are conducted following a standardized process involving the research team, the SBEA and the internal EC. First, meetings between the SBEA and the researcher are organized to discuss the trial design and the possibility of replacing live animals with artificial models. The project authorization form is then collaboratively completed, and the legislative recommendations in terms of animal reduction and experimental procedure refinement are acknowledged. To comply with the reduction principle, the IRSEA relies on an internal statistical service whose task is to decide the correct sample size. Concerning refinement, the best practices are suggested to minimize overall animal stress and suffering. Appropriate techniques to lift, transport and restrain animals are therefore selected for each species. For example, in studies with rabbits, it is recommended that animals be held and carried in the arms, while injections can be done by wrapping the animal with a towel to avoid struggle [52]. In cat studies, it may be suggested to allow an animal to spontaneously exit its carrier instead of forcing it out or, alternatively, to remove the cover from the carrier and handle the cat inside it [53]. In addition to these measures, programs aimed at helping animals become accustomed to a new testing environment may be recommended to avoid novelty-related stress. This point is important for both welfare reasons and research outcomes. Indeed, if the study aims to assess the interaction of the animal with a specific device, prior habituation to the test room is important to increase the chances of observing the desired behaviors instead of seeing the animal spend time exploring. Training sessions related to experimental procedures are also planned to prevent physiological and behavioral stress-related reactions. While many of these activities are part of the daily caregivers’ mission (e.g., animal training for blood collection), others may be study-specific and added to the staff schedule. It is important to mention that the SBEA’s advice must align with the research needs; for example, if the animal’s reaction to a new room is the behavior to be assessed, a habituation session is not considered. Further suggestions may relate to the anesthesia and analgesia protocols and the appropriate recovery follow-up. All decisions are approved by the research team and defined in the project authorization form before being submitted for ethical evaluation. If changes are required for final authorization, the SBEA is further involved to support the researcher in adapting the protocol, and meetings with the EC can be organized to better adhere to its expectations. Before projects start, the SBEA’s mission includes a competence assessment of all personnel involved in the conduct of the procedures, followed by training sessions if needed. During experiments, the SBEA ensures compliance with the validated strategies while being available to deal with unforeseen issues. Once studies are completed, the SBEA’s functions are aimed at monitoring the animals involved and evaluating the effects of the experiments on them; meetings with the research staff can also be planned as a time to reflect on the work done and on further improvements. Communication and knowledge sharing between those conducting the experiments and those caring for the animals are encouraged to inform caretakers about the research goals and help them feel involved and motivated. Finally, relations with the EC are maintained throughout the project to share information on animal welfare matters, explain the research needs and agree on the overall decisions. This teamwork among the SBEA, EC and research personnel allows the IRSEA to achieve important goals related to the three Rs. In addition to efforts to obtain social and collaborative animals through refining strategies, replacement practices are also implemented when possible. An example is the artificial feeding technique for a laboratory mosquito colony [54]. Since adult females must have a blood meal for egg production, live hosts such as guinea pigs are often used. The costs of the animals’ maintenance and the ethical debate around their use pushed the insect research team to assemble and adopt a homemade and low-cost device able to provide blood and completely replace the research animals.

### 4.4. The IRSEA’s SBEA: Challenges and Perspectives

Although the SBEA in our case study appears to be efficient, its role is nevertheless challenging. Initially, much communication was needed to introduce the SBEA to staff, spread its ethical framework and explain its upcoming actions. The education of caregivers in animal handling, socialization and training programs continues to be one of the most energy- and time-consuming objectives. Here, the major challenge for the SBEA is to ensure proper implementation of its requirements by the personnel while regularly evaluating their skills and their results. In relation to project applications, filling in the authorization forms appropriately may require considerable effort, especially when the SBEA’s advice on experimental practices does not align with the needs of researchers. Regarding compliance with the three Rs, while achievements have been made on refinement measures, the strategies for reduction and replacement still need to be improved. Faced with these issues, some solutions have been implemented, while others are currently being studied. Since the efficiency of the SBEA relies on clear and timely communication, various channels, such as meetings, emails and conferences, are used to keep all staff informed on animal welfare matters. For better staff training, schedules defining the nature of SBEA activities, their weekly distribution and the staff involved are present in all facilities. When disagreements arise with researchers, the SBEA asks the EC to intervene for further advice; if necessary, the SBEA is entitled to also request the intervention of the national committee, even though no such situation has arisen so far. Looking ahead, as the SBEA evolves over time, the inclusion of other members with different types of skills could improve current performance; in particular, the establishment of a group of experts on the three Rs could be useful to move forward in applying and developing them.

## 5. Discussion

Regulatory frameworks for the protection of laboratory animals are still highly heterogeneous across the globe [38]. Alongside strict government laws, smaller systems based on institutional guidelines can sometimes be responsible for monitoring animal use [21]. However, countries that are lagging behind in terms of legislation are making progress to move closer to Western international standards with the aim of adopting practices that are acceptable within the community [19,21]. The three Rs ethical principle was developed over 50 years ago, and since then, it has represented the common denominator of legislative programs. Its implementation demonstrates the global trend to enhance quality science and animal welfare through more judicious procedures [10]. In this legal landscape, the European laboratory animal care and use environment is considered one of the most strictly regulated in the world [38]. This reputation is based on modern policies and oversight structures, such as AWBs, that were established to give animal welfare the highest priority in the context of animal keeping, breeding and use [36]. According to the European Commission Report of 2020 [9], the AWB is indeed recognized as a very positive step to improve welfare and science. To help member states put guidelines into practice and ensure a common understanding, the European Commission produced guidance documents [36] to clarify the functioning and mission of the AWB. However, despite this support, the numerous responsibilities that the EU directive gives AWBs can appear complex. The type of involvement in project authorization, the nature of interactions with ECs and the content of staff training are just some of the aspects to be defined and harmonized [42]. All these points are analyzed here by comparing what the law requires and what its practical instantiation could be. Since the French system is well organized and efficient, the authors decided to take it as the reference case for the practical section of the manuscript. We therefore describe the SBEA example, including the administrative aspects of and concrete feedback on the application of the three Rs. The particularity of this SBEA is the members’ knowledge of animal welfare topics as part of the scientific programs of the research institute. While this knowledge can facilitate the overall functioning and achievement of goals of the SBEA, at the same time, it gives the structure the responsibility to actively participate in scientific, ethical and legal advancements on the protection of laboratory animals [55]. Concerning the membership, the choice of the SBEA to include a veterinarian and the director of the institute allows it both to keep itself regularly informed on animal health and to have some authority over the decisions to be made. Additionally, the fact that the members are all research department heads aligns with the legal suggestion of having people with influencing and educational skills. As there are no legal limits on the number of members, the inclusion of multiple profiles could enrich the SBEA’s competences and make it more efficient. The nature of SBEA interactions with supervisory bodies is suggested, but no personal feedback is available to describe either communication with these bodies or with the national committee. Although this point deserves to be expanded upon, the relationship with the internal EC is carefully analyzed, as it represents an important and nonuniform aspect of EU legislation. Although in France the evaluation of experimental projects is assigned to ECs [13], our example shows how AWBs can provide the research project manager with useful help on authorization forms and compliance with the three Rs, thus facilitating the approval procedure. In fact, while the three Rs appear simple, they can sometimes be difficult for researchers to understand [2,29,56]. Since the protocols are reviewed in advance to align with ethical requirements, verification by the EC of projects’ compliance is thus facilitated. The core of the article is dedicated to discussing the SBEA’s commitment in the application of the three Rs to all phases of animal life. Since the IRSEA’s research is mainly focused on behavioral studies, in which animal replacement and reduction are difficult to apply, refinement activities represent the major point to address. Accurate description of behaviors requires good subjects; appropriate housing systems and specific enrichment programs are thus crucial to maintaining the research animals’ physical and mental health [57]. In addition, habituation to personnel, handling and experimental settings helps animals better cope with stressful situations arising later in studies and during routine care [58]. While an association between laboratory procedures and stress responses has been documented [47], friendly handling may, in contrast, favor a positive state, as suggested by Hurst (2010), who observed that mice manipulated using tubes or cupped hands are much less stressed than tail-picked mice [59]. Concerning environmental enrichment, although research is still progressing to find validated scientific measures to assess its effects on animals [60] it is logical to relate it to improved welfare. Training activities with the use of positive reinforcement are a good example of refinement since animals are given the opportunity to cooperate while being rewarded, thus reducing the need for restraints or sedation [56,61]. To meet the directive goal of establishing new refinement strategies, the SBEA could also promote other conditioning methods, such as the use of clickers, to both improve the training of animals and offer them more cognitive enrichment [62]. If refining activities are essential for improving the lives of captive animals, their application can at the same time play a major role in optimizing facility management and scientific studies. Since social activities create daily contact between caregivers and animals, health problems are promptly detected, allowing the veterinarian to intervene quickly and in better conditions. As a result of training sessions, animals are more social and collaborative, which makes handling safer for themselves and for staff. Easier procedures require fewer personnel, freeing up staff who can then be assigned to other tasks; furthermore, as the animals are trained, activities become faster and the remaining time can be invested differently. Regarding personnel, enrichment programs are able to reward caregivers and create a more pleasant and manageable work situation [30]. The social activities fostered in the institute are in fact able to create a good human–animal relationship and an ideal environment where the experiences of each are as positive as possible and able to reciprocally affect their welfare. Moreover, as the skills acquired by employees allow them to become increasingly autonomous, some can in turn be designated to teach new employees, leaving the SBEA in a guidance role. Regarding the effects on research, the benefits of applying refinement for the quality of results can be considerable. In fact, while proper husbandry and care can reduce the stress response that may confound research outcomes [33], training of personnel and animals in procedures allows more efficient and homogeneous work. Finally, the application of refinement strategies may indirectly allow the institute to meet the reduction principle. Indeed, as the animals become trained and valuable, the need for euthanization or rehoming at the end of a study are minimized, and animals can be used again, thereby reducing their overall number [58]. Given all its aims, we agree with Grignaschi et al. (2018) [42] when they say that the AWB, or in this case the SBEA, can be considered as doing a real “service” to the organization that sets it up. Although the AWB has a serious role in adherence to legal requirements, this should never be considered an obstacle but instead a beneficial tool to guarantee high standards of animal welfare in accordance with research needs.

## 6. Conclusions and Perspectives

The international trend toward harmonizing animal care and use practices goes hand in hand with the improvement of regulations aimed at supervising human research. Although minimum standards for breeding and procedures are commonly provided by laws, application of these standards can often be a necessary but nonetheless insufficient condition for ensuring the welfare of laboratory animals. Adoption of the three Rs by the scientific community has addressed concerns over animal protection and stimulated progress in the development of alternative and more refined methods [29]. AWBs are the administrative tools at the forefront of the safeguarding of research animals. The way that they are organized is still being adjusted to the relevant legal provisions, so the French example analyzed here could add information and provide some ideas for the functioning of other bodies. The authors are aware that the model reported could appear to offer a real “service” because of the members’ heavy workload. It was the choice of the French research institute to invest in a complex system based on the spread of a culture of care and ethical values for all scientific personnel. In fact, because all workers, including caretakers and researchers, are responsible for the animals that they deal with, the welfare of laboratory species can be protected only with an effective work team. In particular, it has been shown how positive human–animal interactions—one of the key aspects of the SBEA’s goals—are refined and encouraged. This approach promotes emotional bonds between animals and staff, who feel more motivated to provide high-quality care, with benefits for animal welfare, the work environment and the validity of the study results [63,64]. In light of these considerations, laboratory animals should gain more esteem and not simply be “used” in studies but be considered real “workers”, with needs and rights to be respected. The growing research on animal emotions and cognitive capacities should highlight the moral status of laboratory animals and lead institutions to make advancements in ethical practices [65] while ensuring that animals have lives worth living. Much can still be done to spread this ethical view, and it is hoped that manuscripts such as this one and the other contributions in this issue can show the commitment of the scientific community in this area to improve both animal welfare and research results.

## Data Availability

Data sharing not applicable.
